# Phenotype Differences Between ATP13A2 Heterozygous and Knockout Mice Across Aging

**DOI:** 10.3390/ijms26157030

**Published:** 2025-07-22

**Authors:** Kristina Croucher, Josephine K. Lepp, Jennifer Bechtold, Edward J. Hamad, Sophia Scott, Christian Bittner, Sara Rogers, Christian Ong, Shannon Boehme, Zhuo Wang, Li Lin, Xinwen Wang, Sheila M. Fleming

**Affiliations:** Department of Pharmaceutical Sciences, Northeast Ohio Medical University, Rootstown, OH 44272, USA; kcroucher@neomed.edu (K.C.); jlepp@neomed.edu (J.K.L.); jbechtold@neomed.edu (J.B.); ehamad@neomed.edu (E.J.H.); sscott2@neomed.edu (S.S.); cbittner@neomed.edu (C.B.); srogers3@neomed.edu (S.R.); cong@neomed.edu (C.O.); sboehme@neomed.edu (S.B.); zwang1@neomed.edu (Z.W.); llin@neomed.edu (L.L.); xwang2@neomed.edu (X.W.)

**Keywords:** Parkinson’s disease, alpha-synuclein, polyamines, sensorimotor, cognition

## Abstract

ATP13A2 is a lysosomal polyamine transporter with loss of function mutations linked to multiple neurodegenerative disorders including Parkinson’s disease (PD). Knockout of ATP13A2 in mice leads to age-related sensorimotor impairments and in the brain lipofuscinosis, gliosis, and modest alpha-synuclein (αSyn) pathology. However, few studies have included ATP13A2 heterozygous mice as a comparison. In the present study, the effect of reduced or complete loss of ATP13A2 function on behavior, αSyn, gliosis, dopamine, and polyamines were determined in mice. Male and female ATP13A2 wildtype (WT), heterozygous (Het), and knockout (KO) mice were assessed behaviorally at 3, 12, and 18 months of age. In the brain, αSyn, phosphorylated αSyn, and GFAP were measured in the prefrontal cortex, striatum, ventral midbrain, and cerebellum. Polyamine and neurotransmitter analyses were performed in the same brain regions. Similar to previous studies, KO mice developed motor impairments and widespread gliosis in the brain. In addition, polyamine content was altered in Het and KO mice. In contrast, Het mice showed impairments in cognitive function and an age-related increase in αSyn in the brain. These results indicate potentially different pathological mechanisms when ATP13A2 is reduced compared to when it is knocked out and may have important implications for disease modification in synucleinopathies including PD.

## 1. Introduction

ATP13A2 is a P5-type ATPase, part of a large family of proteins involved in the transport of cations and other substrates across cell membranes through the utilization of energy from ATP hydrolysis [1,2]. P5-type ATPases includeTP13A1-ATP13A5 and are the least characterized of the P-type ATPases. They are only expressed in eukaryotes, and of the P5-types, ATP13A2 is most abundant in the brain [1]. Loss of function mutations of ATP13A2 are linked to multiple neurodegenerative conditions including Kufor-Rakeb Syndrome (KRS; PARK9), an autosomal recessive form of Parkinson’s disease (PD), early onset PD, Neuronal Ceroid Lipofuscinosis (NCL), complicated hereditary spastic paraplegia, and neurodegeneration with brain iron accumulation [3,4,5,6]. Genetic analysis also shows that ATP13A2 variants are frequent in Leucine-rich kinase 2 (PARK8) G2019S carriers, the most common cause of hereditary PD, and may modify disease onset and severity [7]. In idiopathic PD and Dementia with Lewy Bodies, ATP13A2 protein levels are significantly decreased postmortem, suggesting that altered ATP13A2 function may be more pervasive in phenotypic PD than previously considered [8]. While there is a certain amount of clinical heterogeneity between the different ATP13A2-related disorders, they all have symptoms associated with basal ganglia and/or nigrostriatal dysfunction in common [3,9,10,11]. For example, KRS can present with bradykinesia, rigidity and cognitive dysfunction, NCL can include PD-like stooped posture, shuffling gait, and bradykinesia, and some cases of hereditary spastic paraplegia present with resting tremor and akinesia that are responsive to levodopa [5,6,12]. To date, there is only one autopsy case of KRS that shows loss of pigmented neurons in the substantia nigra, lipofuscin accumulation in multiple brain regions, iron accumulation in basal ganglia, and temporal lobe atrophy [13].

ATP13A2 is located on late endosomes and lysosomes and is highly expressed in neurons in the ventral midbrain including the substantia nigra, but it is also found in basal ganglia structures (globus pallidus and putamen), hippocampus, cortex, and the cerebellum [3]. While it remains unclear exactly how ATP13A2 dysfunction leads to neurodegeneration, early in vitro studies in yeast, dopaminergic cells, and iPSCs show ATP13A2 dysfunction impacts lysosomal and mitochondrial function and when overexpressed can protect against alpha-synuclein (αSyn), a key protein in PD, and heavy metal toxicity [14,15,16,17].

Intracellularly, ATP13A2 is involved in the export of polyamines from the lysosome to the cytosol [18,19,20]. Polyamines (putrescine, spermidine, and spermine) bind to nucleic acids and aid in gene transcription and translation, cell cycle progression, oxidative stress response, and metabolism (For review see [21,22]). While in vitro and biochemical studies show accumulation of polyamines in the lysosome when ATP13A2 function is disrupted, it is still unclear how this manifests in an in vivo system.

In mice, global knockout of ATP13A2 results in gliosis, αSyn and ubiquitin accumulation, lipofuscin deposition in the brain, autophagy defects in the liver, and sensorimotor deficits [2,23]. In addition, ATP13A2 knockout mice show an increased sensitivity to manganese and an exacerbation of motor defects when crossbred with αSyn overexpressing mice [24,25]. More recently, work in adult non-human primates and mice shows that loss of ATP13A2 function specifically within the nigrostriatal system results in degeneration of dopamine neurons, strengthening the link between ATP13A2 and PD [26,27]. The study in non-human primates also found an increase in phosphorylated αSyn supporting an interaction between ATP13A2 dysfunction and αSyn pathology [27]. However, it is not known how polyamine homeostasis is affected in vivo.

The present study sought to examine the effect of reduced or complete loss of ATP13A2 function on behavior, αSyn, gliosis, dopamine, and polyamines in vivo across age. Male and female Atp13a2 wildtype (WT), heterozygous (Het), and knockout (KO) mice were assessed for motor and non-motor functions at 3, 12, and 18 months of age. In the brain, αSyn, phosphorylated αSyn, and GFAP were measured in the prefrontal cortex, striatum, ventral midbrain, and cerebellum at the same ages. Polyamine and neurotransmitter analyses were performed in prefrontal cortex, striatum, ventral midbrain, and cerebellum at 3 and 18 months.

## 2. Results

**Figure 1:**
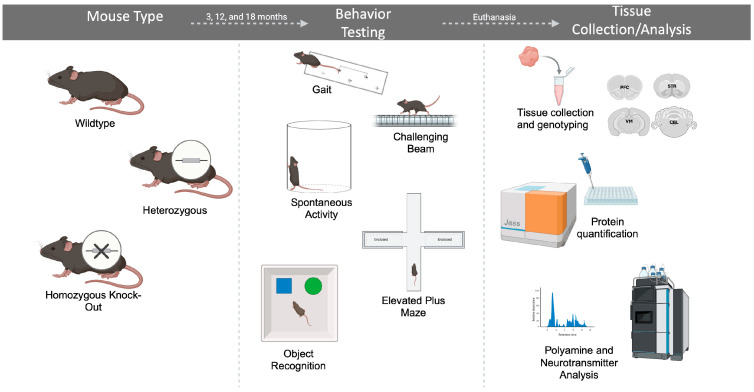
Schematic of experimental design.

Figure 1 shows a schematic of the experimental design for the study.

### 2.1. Behavior

Separate cohorts of mice were assessed at each age. Since the study was not designed to track behavioral changes within the same animal as they age (repeated testing), the main behavioral differences are presented to highlight what differences were detected at each age. Supplemental Data is included to show behavioral scores across age for each genotype (Figures S1 and S2).

**Figure 2:**
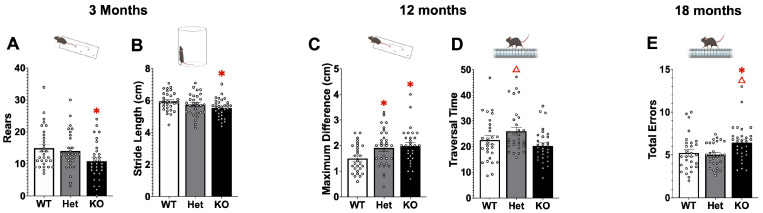
Sensorimotor function was measured in wildtype (WT), Atp13a2 heterozygous (Het) and Atp13a2 knockout (KO) at 3, 12, and 18 months of age. At 3 months (**A**,**B**) KO mice show impairments in gait and in spontaneous activity compared to WT. At 12 months of age (**C**,**D**) both Het and KO display more variable gait compared to WT and Het mice take longer to traverse the challenging beam compared to KO mice. At 18 months of age (**E**) KO mice make more errors on the challenging beam compared to WT. * represents *p* < 0.05 compared to WT mice, Δ represents *p* < 0.05 compared to KO mice. One-Way ANOVA, Tukey’s post hoc.

Sensorimotor deficits were found at each age (Figure 2). At 3 months gait and spontaneous activity were altered in KO mice. For gait, KO mice made significantly shorter strides compared to WT (F[2,87] = 3.86, *p* < 0.05) and in the cylinder KO mice made fewer rears compared to WT (F[2,87] = 3.76, *p* < 0.05). At 12 months differences were observed in gait and the challenging beam. For gait, stride variability (maximum stride difference) was significantly increased in Het and KO compared to WT mice (F[2,86] = 4.54, *p* < 0.05) and on the challenging beam Het mice took significantly longer to traverse the beam than KO mice (F[2,86] = 3.99, *p* < 0.05). At 18 months differences were only identified in the challenging beam with KO mice making significantly more errors than WT and Het mice (F[2,84] = 4.68, *p* < 0.05).

**Figure 3:**
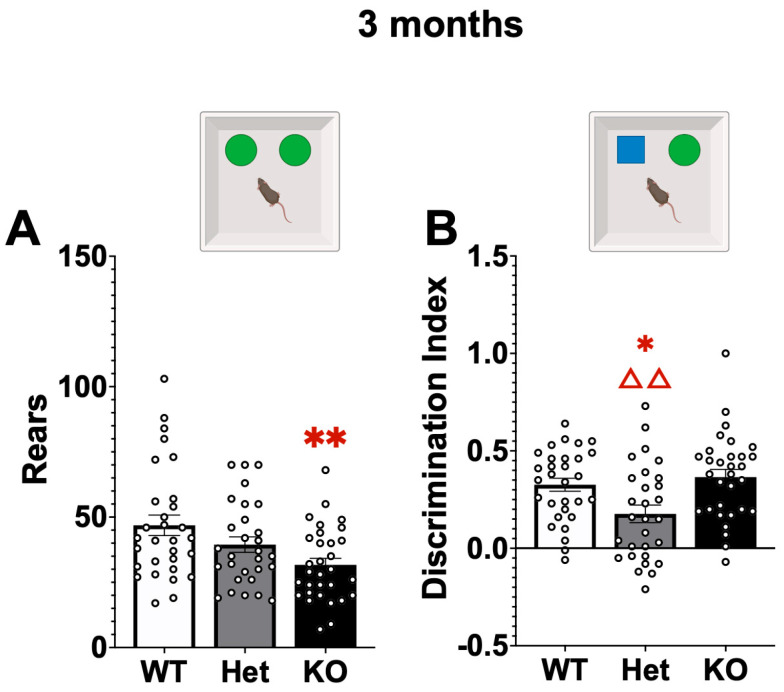
Cognitive function was measured in wildtype (WT), Atp13a2 heterozygous (Het) and Atp13a2 knockout (KO) at 3, 12, and 18 months of age using an object recognition test. At 3 months KO mice reared less than WT mice in the Sample Trial (**A**) and in Het mice the discrimination index (Novel-Familiar Investigation time/Novel + Familiar Investigation Time) was significantly reduced compared to both WT and KO mice (**B**). *, ** represents *p* < 0.05, 0.01, respectively, compared to WT mice, ΔΔ represents *p* < 0.01 compared to KO mice. Mann–Whitney U (Rears), One-Way ANOVA (Discrimination Index), Tukey’s post hoc.

Non-motor function was affected in Het and KO mice at 3 months (Figure 3). In the Sample Trial in object recognition KO mice again made significantly fewer rears compared to WT mice (Mann–Whitney U, *p* < 0.01). Then in the Test Trial the discrimination between a novel and familiar object was significantly decreased in Het compared to WT and KO mice (F[2,87] = 6.28, *p* < 0.01, Tukey’s HSD = *p* < 0.05 compared to WT, *p* < 0.01 compared to KO). No differences were detected in elevated plus maze (*p* > 0.05).

**Figure 4:**
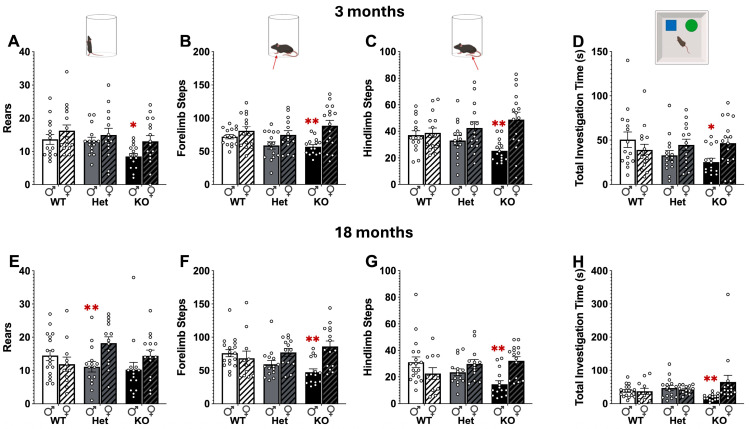
Sex differences were analyzed in wildtype (WT), Atp13a2 heterozygous (Het) and Atp13a2 knockout (KO) mice. At 3 (**A**–**C**) and 18 (**E**–**G**) months, male KO mice were less active in the cylinder test of spontaneous activity compared to female KO mice. Sex differences were also detected in the object recognition test at 3 (**D**) and 18 (**H**) months. Male KO mice spent less time investigating the objects compared to female KO mice. *, ** represents *p* < 0.05, 0.01, respectively, compared to female KO mice. 3 × 2 completely randomized ANOVA, followed by Tukey’s post hoc.

Sex differences were assessed in both motor and non-motor assays. Differences between male and female behaviors were detected in spontaneous activity in the cylinder and in the object recognition test (Figure 4). In the cylinder male KO mice were less active than females at 3 months in number of rears (Main effect of Genotype: F[2,84] = 3.91, *p* < 0.05 and Sex: F[1,84] = 5.35, *p* < 0.05), forelimb steps (Main effect of Sex: F[1,84] = 16.41, *p* < 0.01), and hindlimb steps (Main effect of Sex: F[1,84] = 12.58, *p* < 0.01 and Genotype × Sex interaction: F[2,84] = 3.87, *p* < 0.05). Similarly, at 18 months male KO mice made fewer rears (Genotype × Sex interaction: F[2,81] = 3.56, *p* < 0.05), forelimb steps (Main effect of Sex: F[1,81] = 8.93, *p* < 0.01 and Genotype X Sex interaction: F[2,81] = 6.39, *p* < 0.01), and hindlimb steps (Genotype × Sex interaction: F[2,81] = 7.61, *p* < 0.01) compared to female KO mice. In the object recognition test male KO mice investigated the objects less compared to female KO mice at 3 months (Genotype × Sex interaction: F[2,84] = 3.48, *p* < 0.05) and 18 months (Genotype × Sex interaction: F[2,80] = 3.68, *p* < 0.05).

### 2.2. Real Time qPCR

Tissue analysis using RT-qPCR confirmed varying degrees of *Atp13a2* mRNA expression in WT, Het, and KO tissues from PFC, STR, VM, and CBL (Table 1). Expression levels were highest in WT, lower in Het, and no *Atp13a2* mRNA expression was detected in KO mice.

### 2.3. Protein Quantification

**Figure 5:**
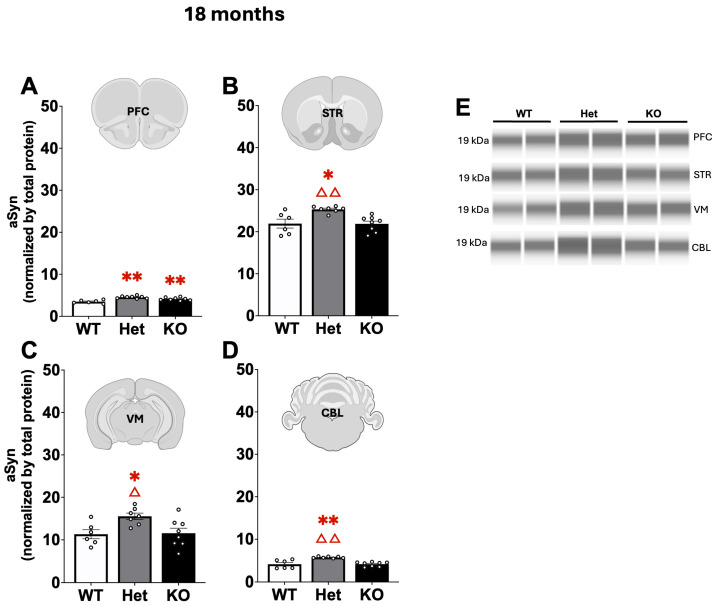
Alpha-synuclein (αSyn) expression in the PFC (**A**), STR (**B**) VM (**C**), CBL (**D**), and representative blots (**E**) at 18 months of age. In the PFC both Het and KO mice had significantly increased αSyn expression compared to WT. In the STR, VM, and CBL αSyn expression was significantly increased in Het mice compared to both WT and KO. *, ** represents *p* < 0.05, 0.01, respectively, compared to WT mice, Δ, ΔΔ represents *p* < 0.05, 0.01, respectively, compared to KO mice. Mann–Whitney U.

Quantification of αSyn, p-αSyn, and GFAP protein in PFC, STR, VM, and CBL was performed using the JESS Simple Western^TM^ system, a capillary based immunoassay. Differences in αSyn expression were found at each age with slight changes at 3 and 12 months (Table 2) and more robust alterations in the older 18 month mice (Figure 5). At 3 months of age αSyn expression in Het mice was significantly increased compared to KO mice in the PFC and CBL (Mann–Whitney U, *p* < 0.01 for both regions; Table 2). Similarly, at 12 months, Het mice had significantly higher αSyn expression in the CBL compared to KO mice (F[2,21] = 4.15, *p* < 05, Tukey’s post hoc; Table 1). However, at 18 months in the PFC both Het and KO mice had significantly increased αSyn expression compared to WT (Mann–Whitney U, *p* < 0.05 for KO, *p* < 0.01 for Het). In the STR, VM, and CBL Het αSyn expression was significantly higher compared to both WT and KO mice (Mann–Whitney U, *p* < 0.05 or *p* < 0.01; Figure 5).

**Figure 6:**
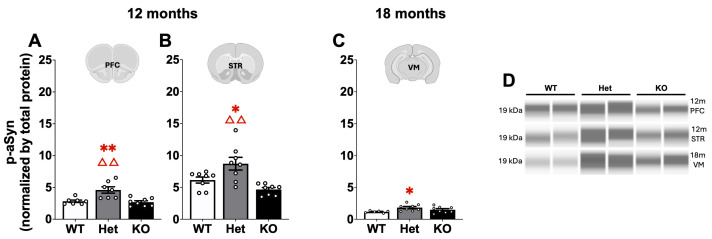
Phosphorylated αSyn (p-αSyn) accumulation is increased in Het mice in multiple brain regions. At 12 months of age p-αSyn was significantly increased in the PFC (**A**) and STR (**B**) in Het mice compared to both WT and KO mice. At 18 months p-αSyn was significantly increased in the VM (**C**) in Het mice compared to WT, (**D**) representative blots. *, ** represents *p* < 0.05, 0.01, respectively, compared to WT mice. ΔΔ represents *p* < 0.01 compared to KO mice. Mann–Whitney U.

Similarly, differences in p-αSyn expression were found at each age with slight changes at 3 months (Table 3) and more robust alterations in the older 12–18 month mice (Figure 6). Expression of p-αSyn was primarily altered in Het mice. At 3 months, p-αSyn expression was significantly increased in Het compared to KO mice in the STR (Mann–Whitney U, *p* < 0.01; Table 3). At 12 months of age, p-αSyn expression was significantly higher in Het PFC and STR compared to both WT and KO mice (Mann–Whitney U, *p* < 0.05 or *p* < 0.01; Figure 5). At 18 months in the VM, p-αSyn expression was increased in Het compared to WT mice (Mann–Whitney U, *p* < 0.05; Figure 6).

**Figure 7:**
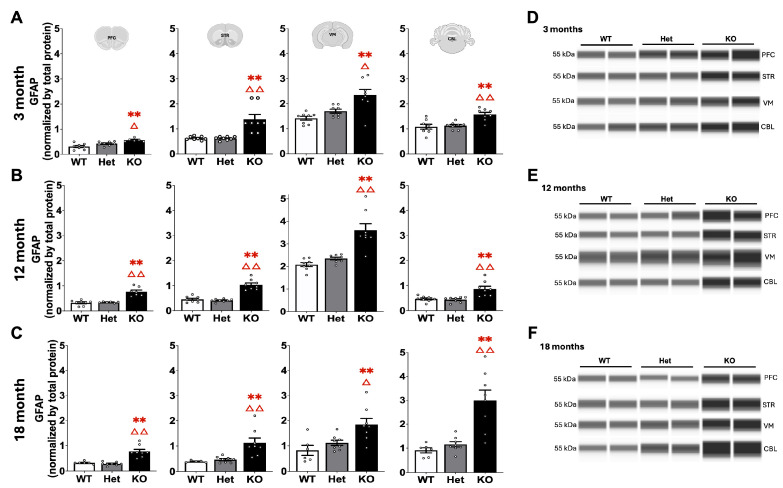
GFAP expression in the PFC, STR, VM, and CBL at 3 (**A**), 12 (**B**), and 18 (**C**) months of age with representative blots (**D**–**F**). ** represents *p* < 0.01 compared to WT mice, Δ, ΔΔ represents *p* < 0.05, 0.01, respectively, compared to Het mice. Mann–Whitney U.

Consistent with previous studies, GFAP was robustly increased in KO mice (Figure 7, [23]). In all brain regions and at each age KO mice had significantly higher expression of GFAP compared to WT and Het mice (Mann–Whitney U, *p* < 0.01).

### 2.4. Mass Spectrometry

**Figure 8:**
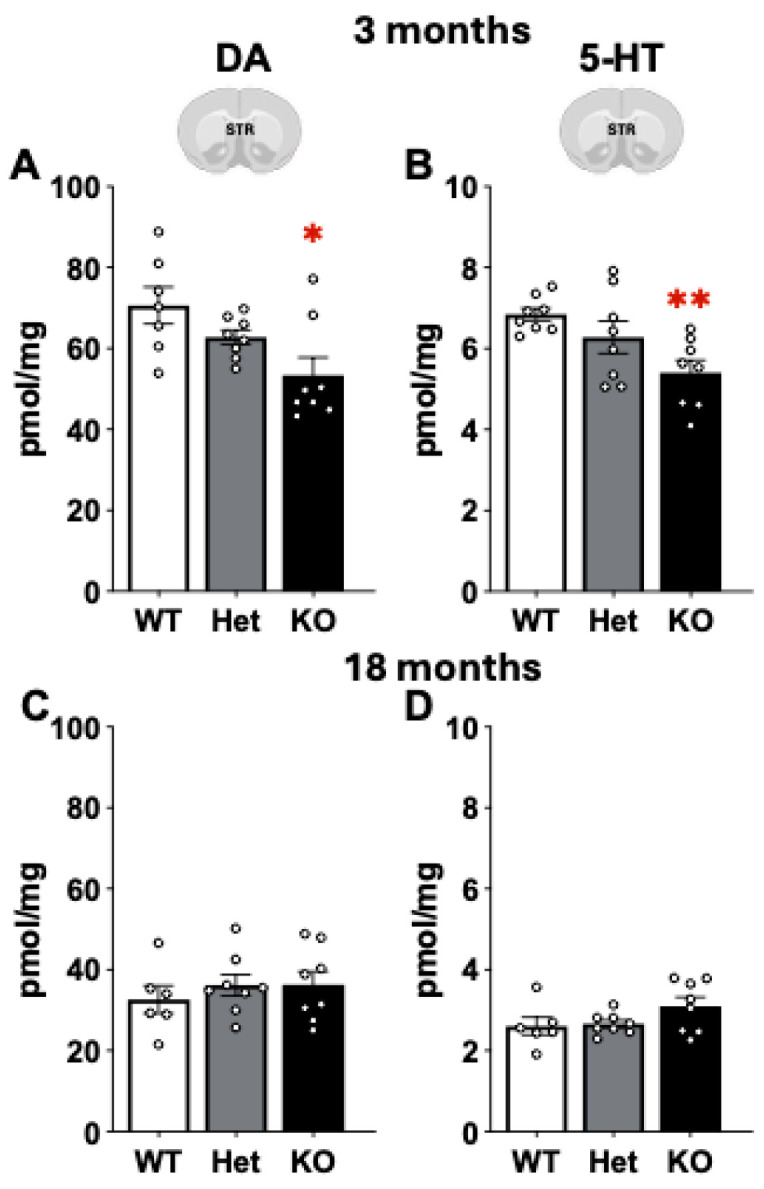
Dopamine (DA) and serotonin (5-HT) content were measured in the striatum in WT, Het, and KO mice at 3 (**A**,**B**) and 18 months (**C**,**D**) of age. Both neurotransmitters were significantly decreased in KO mice compared to WT at 3 months of age. *, ** represents *p* < 0.05, 0.01, respectively, compared to WT mice but not at 18 months. One-Way ANOVA followed by Tukey’s post hoc (DA) and Mann–Whitney U (5-HT).

Mass spectrometry was used to measure the neurotransmitters dopamine and serotonin and the polyamines putrescine, spermidine, and spermine at 3 and 18 months of age. Differences in dopamine and serotonin content were detected in KO mice (Figure 8). At 3 months dopamine was significantly decreased in the STR in KO compared to WT (F[2,20] = 5.28, *p* < 0.05, Tukey’s post hoc). Similarly, serotonin levels were significantly lower in KO compared to WT mice (Mann–Whitney U, *p* < 0.01). Changes in DA and 5-HT were not correlated with behavior (*p* > 0.05). No differences in neurotransmitter content were observed at 18 months of age.

**Figure 9:**
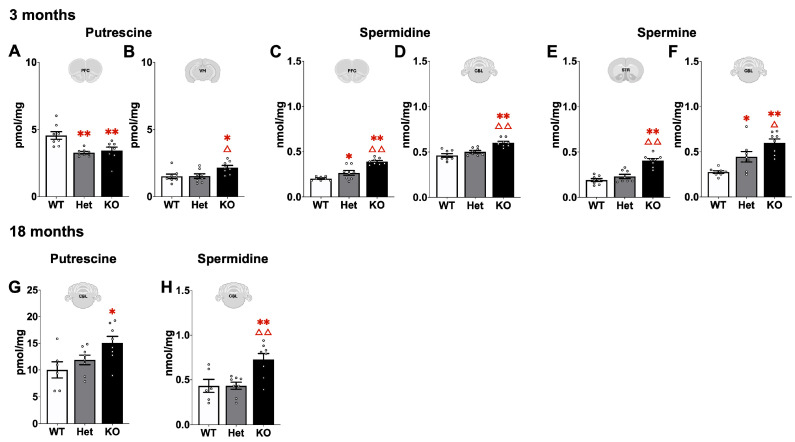
Polyamine analysis was performed in the PFC, STR, VM, and CBL at 3 and 18 months of age. At 3 months, differences were detected in putrescine in the PFC (**A**) and VM (**B**), spermidine in the PFC (**C**) and CBL (**D**), and spermine in the STR (**E**) and CBL (**F**). At 18 months of age differences in putrescine (**G**) and spermidine (**H**) were detected in the CBL. *, ** represents *p* < 0.05, 0.01, respectively, compared to WT mice, Δ, ΔΔ represents *p* < 0.05, 0.01, respectively, compared to Het mice. Mann–Whitney U.

Polyamine content was altered in the brain at both ages (Figure 9). At 3 months putrescine was significantly decreased in the PFC in both Het and KO mice (Mann–Whitney U, *p* < 0.01). While in the VM putrescine was increased in KO mice compared to WT and Het (Mann–Whitney U, *p* < 0.05). Spermidine was significantly increased in 3 month KO mice in the PFC and CBL (Mann–Whitney U, *p* < 0.01). For spermine, significant increases were detected in STR in KO mice compared to both Het and WT (Mann–Whitney U, *p* < 0.01) and in the CBL spermine was increased in Het and KO mice compared to WT (Mann–Whitney U, *p* < 0.05 or *p* < 0.01). At 18 months putrescine and spermidine were significantly increased in KO mice in the CBL (Mann–Whitney U, *p* < 0.05, *p* < 0.01).

## 3. Discussion

To date, multiple homozygous and heterozygous mutations in ATP13A2 that interfere with its function have been identified in clinical populations [3,5,6]. These mutations are linked to several neurodegenerative conditions including Parkinson’s disease. While the transport function of ATP13A2 has been elegantly shown, it is still unclear how impaired ATP13A2 leads to neurodegeneration and what specific brain regions are primarily affected [18,19,20]. The present study sought to determine how reduced or loss of function of ATP13A2 across aging impacts behavior, αSyn accumulation and pathology, and polyamine status in different brain regions including the prefrontal cortex, striatum, ventral midbrain, and cerebellum. *Atp13a2* expression was validated in three month WT, Het, and KO mice in each brain region. In all brain regions WT mice showed the highest expression of *Atp13a2* followed by Het with less and no expression was detected in KO mice. As previously shown, global knockout of ATP13A2 in mice results in motor deficits and gliosis across multiple brain regions [2,23]. In addition to this established phenotype, it is now shown that ATP13A2 KO mice display some sex-dependent behavioral deficits, develop early changes in polyamine content in all brain regions examined, and show early changes in neurotransmitter content in the striatum. In contrast, ATP13A2 Het mice develop a different phenotype that includes early cognitive deficits and age-related accumulation of αSyn in multiple brain regions. These results suggest different mechanisms may drive dysfunction depending on whether ATP13A2 is reduced or knocked out.

Previous studies assessing only WT and KO mice show motor deficits in aged KO mice [2,24]. The present study expanded on the behavioral analyses by including Atp13a2 Het mice, more discrete ages rather than grouping animals by age range (i.e., 9–12 months), and by increasing the n-size to be powered to determine potential sex differences. Similar to the earlier studies, KO mice showed sensorimotor deficits in gait, spontaneous activity, and motor performance and coordination [2,24]. Sex differences were also detected in sensorimotor function in KO mice. Male KO mice were less active in the test of spontaneous activity compared to female KO mice. There were motor deficits in Atp13a2 Het mice but overall, they were more modest compared to KO with subtle deficits in gait and motor performance and coordination at 12 months of age.

Mice were also tested on assays that measure cognitive function and emotional reactivity. Early deficits were detected in the object recognition test with KO mice rearing less than WT in the first phase of the test. Sex differences were also found in the object recognition test where male KO investigated the objects less than female KO suggesting impaired attention in male KO mice. Het mice, on the other hand, showed a different cognitive phenotype with more memory-related impairments in object recognition. The deficits in Het mice were transient though and not observed at the older ages. It is possible that this phenotype represents and early subclinical stage of disease.

The pathological accumulation of αSyn, a key protein in the pathogenesis of PD, has been implicated in ATP13A2 dysfunction with studies in yeast and cell systems showing loss of function of ATP13A2 causes increased αSyn toxicity and (endo)lysosomal defects [14,15,16,17]. In the present study, the most robust increase in αSyn expression was observed in older Het mice. Further, phosphorylated αSyn expression (S129), a marker for pathological αSyn, was also increased more robustly in older Het mice. Prefrontal cortex, striatum, and ventral midbrain were affected in Het mice, while KO mice only showed an increase in αSyn in the prefrontal cortex. This suggests that reduced ATP13A2 function may significantly impact αSyn more than a complete loss of ATP13A2. Indeed, recent work in nonhuman primates show that an ~60% knockdown of ATP13A2 expression within the substantia nigra results in increased αSyn pathology and nigrostriatal cell loss [27]. This is important given that the one KRS postmortem case examined did not identify Lewy body pathology and αSyn pathology is not consistently shown in knockout mice [2,13,23]. It is not clear whether there are compensatory mechanisms in KO mice that prevent broad αSyn pathology, although it has been shown that there is no change in other P5-type ATPases when ATP13A2 is knocked out [2]. Compared to other lysosomal mutations linked to PD, the αSyn phenotype in Het mice does resemble that observed in some GBA-related Gaucher models [28]. Taken together, these findings indicate potential different mechanisms of dysfunction depending on whether ATP13A2 function is reduced or completely knocked out. Why Het and KO mice show a different αSyn phenotype is unclear but current work is now focused on assessing autophagy and mitochondrial function in aging WT, Het, and KO mice.

While αSyn pathology was primarily seen in Het mice, a robust increase in GFAP was observed in KO but not in Het mice in the present study. GFAP was increased in all brain regions studied and at all ages in KO mice and is consistent with previous studies in both global and AAV-Cre knockout models [23,29]. However, the lack of effect on GFAP in Het mice differs from important work from Lewis and colleagues [30]. In that study, Het mice at 18 months of age showed increased GFAP immunostaining in cortex, hippocampus, and brainstem. The present study used Western blot and focused on different brain regions which likely accounts for the difference. Therefore, taken together increased gliosis may be present but more limited in Het than in KO mice.

Functionally, ATP13A2 is involved in the export of polyamines from the lysosome to the cytosol [18,19,20]. In vitro and biochemical studies show accumulation of polyamines in the lysosome when ATP13A2 function is disrupted, however it is unknown how this translates in an in vivo system. Therefore, the polyamines putrescine, spermidine, and spermine were measured in multiple brain regions in young (3 months) and older (18 months) mice. All three polyamines were disrupted in multiple brain regions in the young mice. The effects were more pronounced in KO mice, but Het mice also showed modest changes in the prefrontal cortex and cerebellum. At 3 months of age, putrescine, the main precursor to spermidine and spermine, was decreased in the prefrontal cortex in both Het and KO mice and increased only in KO mice in the ventral midbrain. Similarly, spermidine was increased in both Het and KO mice in the prefrontal cortex and increased in just KO in the cerebellum at the same age. While spermine was increased in KO mice in the striatum and in both Het and KO mice in the cerebellum. At 18 months, effects were observed only in cerebellum with putrescine and spermidine significantly increased compared to WT. Thus, polyamine levels were mostly increased in KO mice. In general, polyamine levels are tightly balanced through intracellular metabolism and transport where too much or too little can disrupt proper cellular functioning [22]. Polyamine dyshomeostasis is shown to impact mitochondrial function, autophagy, and inflammation, all factors implicated in neurodegenerative diseases including PD [31,32,33]. There are also cell-type specific differences in polyamine synthesis and transport with neurons linked to synthesis and glial cells with uptake and storage [34]. The robust increase in GFAP in KO mouse brains suggests glial cells may be primarily affected in the present study but this remains to be determined. It is possible that reduced ATP13A2 has a bigger impact on neuronal polyamine balance while complete loss of ATP13A2 has the bigger impact on glial polyamine homeostasis.

Overall, this study reveals different behavioral and pathological phenotypes between Atp13a2 heterozygous and knockout mice as they age. The findings are potentially impactful because aging is the greatest risk factor for synucleinopathies, including PD, and identifying factors that may modulate disease progression is important for therapeutic discovery. The results suggest that reduced expression of ATP13A2 is associated with the development of αSyn pathology and that a complete knockout of ATP13A2 disrupts polyamine homeostasis that is associated with robust gliosis. Future studies are aimed at determining the effect of genotype and aging on autophagy and mitochondrial function.

## 4. Materials and Methods

### 4.1. Animals

Animal care was conducted in accordance with the United States Public Health Service Guide for the Care and Use of Laboratory Animals, and procedures were approved by the Institutional Animal Care and Use Committee at Northeast Ohio Medical University. Atp13a2 heterozygous breeders were originally obtained from The Jackson Laboratory (strain 021914; B6N.129S6(Cg)-Atp13a2tm1Pjsch/J) and male and females were bred together to generate wildtype (WT), heterozygous (Het), and homozygous knockout (KO) mice [2,24,25]. The genotype of all WT, Het, and KO mice was assessed at weaning and confirmed at euthanasia by polymerase chain reaction (PCR) amplification analysis of ear and tail tissue. Mice were aged to 3, 12, or 18 months and maintained on a reverse light/dark cycle with lights off at 11am and lights on at 11pm. All mice were provided access to water and standard rodent chow ad libitum throughout the experiment except during behavioral testing procedures.

### 4.2. Behavioral Testing

Motor and non-motor tests were performed on WT, Het, and KO mice at each age. All behavioral procedures were conducted during the dark cycle under red or low light. Motor function was assessed using a gait test, spontaneous activity in the cylinder, and the challenging beam. Non-motor testing included the elevated plus maze test of emotional reactivity and an object recognition test that measures aspects of attention and memory. Tests were performed over a 5-day period in this order, with no more than two tests performed within the same day. The n-sizes for behavior testing ranged from 28 to 31 per genotype per age and there were 14–18 males and 11–16 females per genotype.

*Gait:* Gait was measured for each mouse as previously described [2,25,35,36]. Briefly, animals were trained to walk through a narrow alley leading into their homecage. Once trained, paper was placed along the alley floor and each animal’s hindlimbs were brushed with non-toxic paint (Crayola©, Easton, PA, USA). Animals were then placed at the beginning of the alley. As they walked into their home-cage they left their paw prints on the paper underneath. Stride length and stride variability were determined by measuring the distance between hindlimb prints. Only strides made while continuously walking (no stopping) were included in the analysis. Stride lengths at the beginning and end of the alley were not counted since animals tend to make irregular steps at the beginning and typically stop and make smaller steps just before entering the cage.

*Spontaneous Activity:* Spontaneous activity was measured using a clear cylinder measuring 15.5 cm high and 12.7 cm in diameter [2,25,35]. The cylinder was placed on a piece of glass with a mirror underneath to allow viewing of forelimb and hindlimb movements. Activity was recorded for 3 min and videos were viewed and rated in slow motion by an experimenter blind to mouse age and genotype. The number of rears, forelimb and hindlimb steps, and time spent grooming were determined for each mouse.

*Challenging Beam:* Motor performance and coordination were measured with the challenging beam traversal test as previously described [2,25,35]. The beam consists of four 25 cm sections totaling one meter and constructed from Plexiglas. The four sections decrease in width across the beam starting with 3.5 cm and narrowing by 1 cm increments to a final width of 0.5 cm. Animals were trained to traverse the beam from the widest section to the narrow end and into their homecage across two days for five trials each day for each mouse. On the day of testing, a mesh grid with 1 cm squares of corresponding width was placed over the surface of the beam segments. The grid left a 1 cm space between the grid and the surface of the beam for scoring purposes. Animals were video recorded while traversing the grid-covered beam for five trials and videos were scored for time to traverse, number of steps, and errors. Errors were counted when a limb slipped between or outside of the mesh grid during a forward movement. Errors, number of steps, and time to traverse were scored by an experimenter blind to age and genotype.

*Elevated Plus Maze:* Emotional reactivity was measured using the elevated plus maze [37,38,39]. The EPM is constructed in the shape of a plus sign (38 cm high) with two open arms (5 cm width, 31 cm length), two closed arms with walls (5 cm width, 15 cm high walls, 31 cm length), and a central platform (5 cm width, 5 cm length). The floor of the maze was lined with textured contact paper to increase grip and encourage exploration. Mice were placed in the center of the maze facing an open arm and allowed to explore for 5 min under red light. If a mouse fell off the maze, it was placed in the same location from which it fell. All testing was recorded and then scored by an experimenter blind to age and genotype. A mouse was considered to be in a section of the maze when all four limbs were within the section. The number of rears and time spent in the open, closed, and center sections were determined for each mouse.

*Object Recognition:* Cognitive function was assessed using an object recognition test that measures attention and memory [37,39,40,41]. Mice were habituated to the test arena (40 cm length, 25 cm width, 20 cm height) for 20 min for two days prior to testing. On the day of the test two trials were conducted, a Sample Trial and a Test Trial. During the Sample Trial, two identical objects (wooden cylinders, 4.2 cm tall and 2.5 cm diameter) were placed at one end of the arena. The objects had a rounded top to prevent excessive climbing. Each mouse was then placed in the center of the arena and allowed to explore for 10 min. After 10 min, the mouse was removed and placed back in the home cage for 60 min. For the Test Trial, one of the objects from the Sample Trial was replaced with a novel object (fishing bobber, 4.2 cm tall and 3.5 cm diameter). The novel object was a similar size to the familiar object but differed in texture and shape. Each mouse was again placed in the center of the arena and allowed to explore for 10 min. The test was video recorded and the number of rears and the time spent investigating each object was determined for each mouse by an experimenter blind to mouse age and genotype**.** Investigation was defined as time spent sniffing an object. A discrimination index was calculated (novel-familiar investigation time/novel + familiar investigation time) as an indicator of memory function.

### 4.3. Tissue Analysis

Following behavior testing, mice were euthanized, and brains were rapidly removed. Using a 1.0 mm coronal brain matrix on ice, the prefrontal cortex (PFC), striatum (STR), ventral midbrain (VM), and the cerebellum (CBL) were isolated using the following bregma coordinates: PFC = 2.8–1.6 mm; STR = 1.4–0.2 mm; VM = −2.10–3.8 mm [42,43]. Brain tissue was stored at −80 °C for later processing of protein quantification and mass spectrometry. The n-sizes for protein quantification and mass spectrometry ranged from 21 to 24 mice per age and within each age there were 3–4 males and 2–4 females per genotype.

*Real Time qPCR*: RNA was isolated from 3 month mice from the PFC, STR, VM, and CBL using Trizol (Thermo Fisher Scientific, Waltham, MA, USA) and cDNA was synthesized using 2 μg RNA. Taqman primers for *Atp13a2* (Mm00661379_m1) and Hprt (Mm03024075_m1; Thermo Fisher Scientific, Waltham, MA, USA) were used to determine relative mRNA expression levels using the ΔΔCt method against Hprt as an internal control.

*Protein Quantification:* Protein analysis was performed using the JESS Simple Western^TM^ System (ProteinSimple^®^, Bio-Techne, Minneapolis, MN, USA), an automated Western blot system that performs protein size separation and immunoblotting using capillary based technology [44,45,46]. Samples and reagents were loaded into a separation module plate and proteins from the samples migrate and immobilize in the capillaries. Protein detection with selected primary and secondary antibodies and quantification using total protein was performed in one run using chemiluminescence signals [44]. Alpha-Synuclein (αSyn, 1:25, mouse, BD Biosciences, #610787, Franklin Lakes, NJ, USA), phosphorylated αSyn (p-αSyn S129, 1:25, rabbit, Cell Signaling, #23706, Danvers, MA, USA), and GFAP (1:25, mouse, Invitrogen, MA5-15086, Carlsbad, CA, USA) were measured in fresh frozen whole tissue samples of the PFC, STR, VM, and CBL. Each region was sonicated on ice using the Branson Digital Sonifier at 10% amplitude (Marshall Scientific, Hampton, NH, USA) with 10 μL T-PER (ThermoFisher, #78510, Waltham, MA, USA) containing protease inhibitor (ThermoFisher, #78429) and phosphatase inhibitor (ThermoFisher, #78428, Waltham, MA, USA) per 1 mg of tissue. Tissue was assayed for total protein concentration using the Pierce BCA Protein Assay Kit (ThermoFisher, #23225, Waltham, MA, USA) and samples were diluted to 2.5 μg/μL and stored at −80 °C for subsequent protein quantification. 2–40 kDa Chemiluminescence or Fluorescence Separation Modules were used for αSyn and p-αSyn (αSyn; Bio-Techne, #SM-W012, #SM-FL006, respectively, Minneapolis, MN, USA) and a 12–230 kDa Chemiluminescence Separation Module was used for GFAP (Bio-Techne, #SM-W004, Minneapolis, MN, USA). The plates were loaded with 22–24 samples that included each genotype and one brain region. All protein quantification was normalized to total protein. All antibodies were optimized for protein and antibody concentration prior to the start of the experiment by running samples through protein dilutions (0.5–1.2 μg/μL) and antibody dilutions using the Simple Western Validated Antibody Database (Bio-Techne, Minneapolis, MN, USA) as a guide. Analysis was completed using the Compass for Simple Western 6.1.0 software.

*Mass Spectrometry*: The polyamines putrescine, spermine, and spermidine and neurotransmitters dopamine and serotonin were quantified using liquid chromatography-tandem mass spectrometry (LC/MS/MS) of fresh frozen PFC, STR, VM, and CBL in 3 and 18 month WT, Het, and KO mice as previously described with slight modifications [47]. Briefly, approximately 20 mg of tissue was weighed and homogenized using soft tissue homogenizing zirconium oxide beads (Caymen Chemical, #10402, Ann Arbor, MI, USA) in 6% trichloroacetic acid (TCA, ThermoScientific, #A11156.30, San Jose, CA, USA) and freshly prepared internal standards consisting of spermidine-D6 (Caymen Chemical, #34697), dopamine-D4 (Cayman Chemical, #36309), serotonin-D4 (Caymen Chemical, #29415, Ann Arbor, MI, USA) in the same concentration of 200 ng/mL. Additionally, the standard curve was prepared using 100 μg/μL putrescine (Sigma, #51799, St. Louis, MO, USA), spermidine (Sigma, #85558, St. Louis, MO, USA), spermine (Sigma, #55513, St. Louis, MO, USA), dopamine (Cayman Chemical, #21992, Ann Arbor, MI, USA) and serotonin (Caymen Chemical, #14332, Ann Arbor, MI, USA) which were serial diluted from 1000 ng/mL to 3 ng/mL. Following homogenization, samples were centrifuged at 25,000× *g* for 10 min at 4 °C and 1.5 μL of the supernatant was removed and diluted 1000× with 6% TCA and internal standards for spermine and spermidine derivatization. Samples used for the derivatization of putrescine, dopamine and serotonin were not diluted. 150 μL of each sample was then combined and incubated at 35 °C for 15 min with 800 μL MS grade water (Sigma, #270733, St. Louis, MO, USA), 125 μL 1 M sodium carbonate buffer (pH 9), and 25 μL isobutyl chloroformate (Sigma, #177989, St. Louis, MO, USA) as the derivatization reagent, then centrifuged at 10,000× *g* for 1 min at 4 °C. The supernatant and standard curve samples were subjected to solid phase extraction (SPE) using HLB 1cc (30 mg) extraction cartridges (Waters, #WAT094225, Milford, MA, USA) following the product manual. Samples were dried in the vacufuge plus (Eppendorf, Enfield, CT, USA) at room temperature until dry, reconstituted with 200 μL 50% MeOH, and vortexed at 1500 rpm for 5 min. Following centrifugation at 25,000× *g* for 10 min at 4 °C, 150 μL of supernatant was transferred to a MS vial for LC/MS/MS. Quantification was performed using the Vanquish Ultra Performance Liquid Chromatography (UPLC, ThermoScientific, San Jose, CA, USA) coupled with a Q-Exactive Plus Orbitrap Mass Spectrometer (MS/MS, San Jose, CA, USA). The analytes were separated by an ACQUITY UPLC BEH C18 1.7 µM 2.1 × 100 mm column at a flow rate of 0.2 mL/min at 40 °C. All MS2 ions associated with respective MS1 ion were monitored using parallel reaction monitoring (PRM) under positive mode with respective collision energy (CE). The targeted analytes were then quantified by extracting their specific transitions using the Skyline Software v23.1 (University of Washington, Seattle, WA, USA). The quantified concentration data of polyamines, dopamine and serotonin were normalized by the weight of the brain soft tissue into nmol/mg tissue or pmol/mg tissue.

### 4.4. Statistics

Both parametric and nonparametric statistics were used to compare genotypes for behavior, protein analysis, and mass spectrometry. For behavior, protein quantification, and mass spectrometry separate analyses were conducted for each age group. A one-way ANOVA followed by Tukey’s post hoc was used to compare differences between WT, Het, and KO mice. For sex differences a 3x2 completely randomized ANOVA was used followed by Tukey’s post hoc. When the data did not meet parametric assumptions then nonparametric analysis using Mann–Whitney U was used. Significance was set at *p* ≤ 0.05. All statistics were calculated using StatPlus:mac AnalystSoft Inc. or GraphPad Prism 10.2.3 software for MacOS.

## Figures and Tables

**Table 1:** *Atp13a2* expression in brain at 3 months.

	WT	Het	KO
PFC	1.10 ± 0.17	0.74 ± 0.09	0.00 ± 0.00
STR	1.09 ± 0.13	0.78 ± 0.13	0.00 ± 0.00
VM	1.08 ± 0.14	0.68 ± 0.09	0.00 ± 0.00
CBL	1.02 ± 0.07	0.55 ± 0.03	0.00 ± 0.00

Mean ± SEM relative mRNA expression normalized to brain region, PFC = prefrontal cortex, STR = striatum, VM = ventral midbrain, CBL = cerebellum N = 7–8 per genotype per brain region.

**Table 2:** Alpha-synuclein protein expression at 3 and 12 months.

**3 Months**	**WT**	**Het**	**KO**
PFC	25.61 ± 1.52	27.79 ± 0.51 ΔΔ	21.98 ± 0.73
STR	18.76 ± 1.06	20.17 ± 0.63	17.81 ± 0.72
VM	11.07 ± 0.54	11.30 ± 0.67	8.29 ± 0.65
CBL	3.81 ± 0.24	4.41 ± 0.15 ΔΔ	3.69 ± 0.11
**12 Months**	**WT**	**Het**	**KO**
PFC	22.42 ± 1.42	25.51 ± 1.02	13.54 ± 1.47
STR	27.24 ± 1.62	27.08 ± 0.65	24.30 ± 1.19
VM	10.10 ± 0.42	10.98 ± 0.55	8.64 ± 0.88
CBL	3.03 ± 0.20	3.41 ± 0.11 Δ	2.80 ± 0.13

Mean ± SEM relative protein expression normalized to total protein, PFC = prefrontal cortex, STR = striatum, VM = ventral midbrain, CBL = cerebellum N = 7–8 per genotype per brain region; Δ, ΔΔ represents *p* < 0.05, 0.01, respectively, compared to KO; Mann–Whitney U.

**Table 3:** Phosphorylated alpha-synuclein protein expression at 3 months.

	WT	Het	KO
PFC	5.02 ± 0.43	5.47 ± 0.17	3.31 ± 0.34
STR	4.83 ± 0.24	6.81 ± 0.91 ΔΔ	2.97 ± 0.13
VM	1.90 ± 0.16	2.32 ± 0.24	1.80 ± 0.21
CBL	0.53 ± 0.08	0.60 ± 0.13	0.42 ± 0.06

Mean ± SEM relative protein expression normalized to total protein, PFC = prefrontal cortex, STR = striatum, VM = ventral midbrain, CBL = cerebellum N = 6–8 per genotype per brain region; ΔΔ represents *p* < 0.01 compared to KO; Mann–Whitney U.

## Data Availability

The raw data supporting the conclusions of this article will be made available by the authors on request.

## Supplementary Materials

The following supporting information can be downloaded at: https://www.mdpi.com/article/10.3390/ijms26157030/s1.

## Abbreviations

The following abbreviations are used in this manuscript:

PD	Parkinson’s disease
KRS	Kufor-Rakeb Syndrome
NCL	Neuronal Ceroid Lipofuscinosis
αSyn	alpha-synuclein
p-αSyn	phosphorylated alpha-synuclein
WT	wildtype
Het	heterozygous
KO	knockout
PFC	prefrontal cortex
STR	striatum
VM	ventral midbrain
CBL	cerebellum
DA	dopamine
5-HT	serotonin
PCR	polymerase chain reaction
GFAP	glial fibrillary acidic protein
LC/MS/MS	liquid chromatography-tandem mass spectrometry
TCA	trichloroacetic acid
SPE	solid phase extraction
PRM	parallel reaction monitoring
CE	collision energy
