# Supplementary material for: Phenotype Differences Between ATP13A2 Heterozygous and Knockout Mice Across Aging
Source: Int J Mol Sci. 2025 Jul 22;26(15):7030. doi: 10.3390/ijms26157030 (PMC12346011; doi:10.3390/ijms26157030)
Supplement: Supplementary file 1 [file ijms-26-07030-s001.zip › ijms-3654990-supplementary.pdf]

International Journal of
Molecular Sciences

MDPI

Supplementary Figures

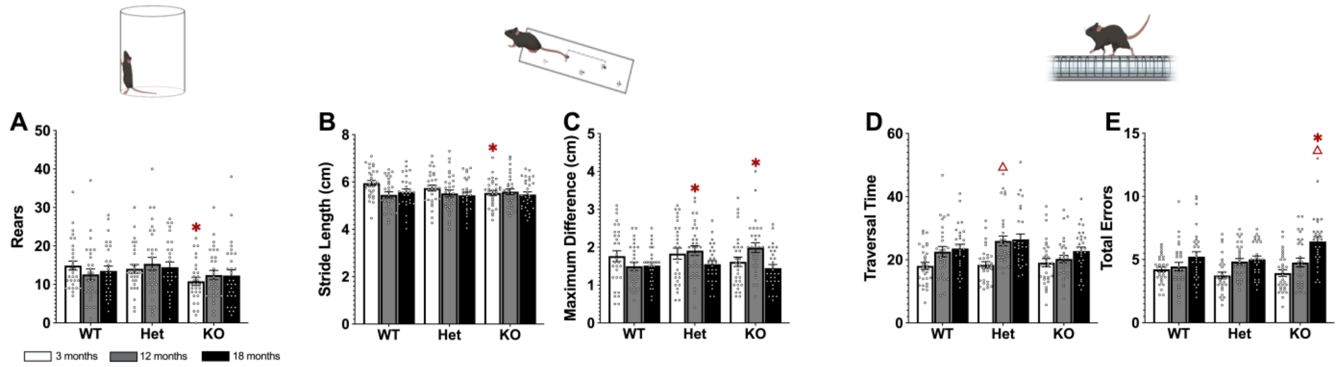

Figure S1. Sensorimotor function was measured in wildtype (WT), ATP13A2 heterozygous (Het) and ATP13A2 knockout (KO) at 3, 12, and 18 months of age. 3 month KO mice show reduced spontaneous activity (**A**) compared to age-matched WT. Impairments in gait were observed in 3 month KO (**B**) compared to age-matched WT and 12 month Het and KO (**C**) compared to age-matched WT. On the challenging beam, 12 month Het (**D**) mice take longer to traverse the challenging beam compared to age-matched WT and 18 month KO (**E**) mice make more errors compared to age-matched WT and Het. * represents p<0.05 compared to WT mice, Δ represents p<0.05 compared to Het or KO mice of the same age. One-Way ANOVA, Tukey's post hoc.

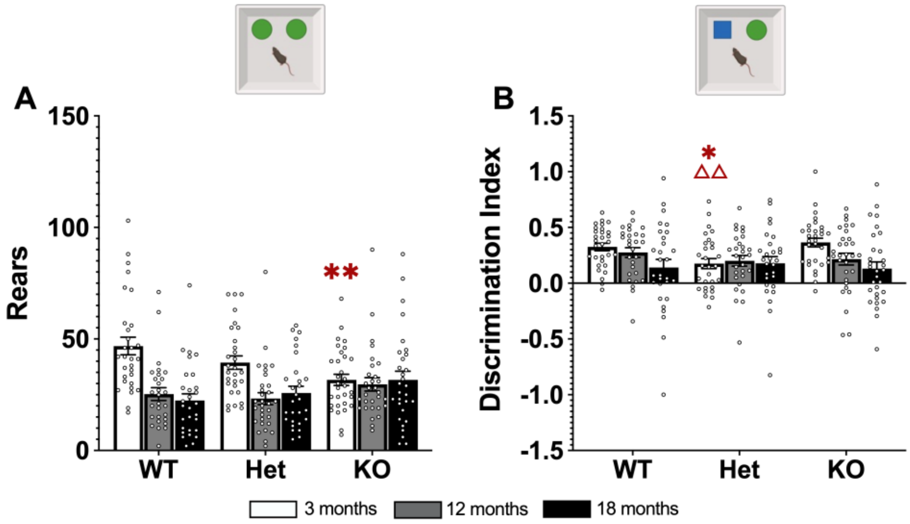

Figure S2. Cognitive function was measured in wildtype (WT), ATP13A2 heterozygous (Het) and ATP13A2 knockout (KO) at 3, 12, and 18 months of age using an object recognition test. At 3 months KO mice reared less than WT mice in the Sample Trial (**A**) and in Het mice and discrimination index was significantly reduced (**B**) compared to both WT and KO mice of the same age. *,** represents p<0.05, 0.01, respectively, compared to age-matched WT mice, ΔΔ represents p<0.01 compared to age-matched KO mice. Mann-Whitney U (Rears), One-Way ANOVA (Discrimination Index), Tukey's post hoc.
